# Artichoke Polyphenols Produce Skin Anti-Age Effects by Improving Endothelial Cell Integrity and Functionality

**DOI:** 10.3390/molecules23112729

**Published:** 2018-10-23

**Authors:** Isabella D’Antuono, Antonietta Carola, Luigi M. Sena, Vito Linsalata, Angela Cardinali, Antonio F. Logrieco, Maria Gabriella Colucci, Fabio Apone

**Affiliations:** 1 Institute of Sciences of Food Production (ISPA), National Research Council of (CNR), Via G. Amendola, 122/O, 70126 Bari, Italy; isabella.dantuono@ispa.cnr.it (I.D.); vito.linsalata@ispa.cnr.it (V.L.); antonio.logrieco@ispa.cnr.it (A.F.L.); 2 Arterra Bioscience srl, Via B. Brin 69, 80142 Napoli, Italy; antonietta@arterrabio.it (A.C.); luigi@arterrabio.it (L.M.S.); gcolucci@arterrabio.it (M.G.C.); fapone@arterrabio.it (F.A.); 3 VitaLab srl, Via B. Brin 69, 80142 Napoli, Italy

**Keywords:** artichoke, polyphenols, vascular aging, cosmetic ingredient, by-products

## Abstract

Artichoke is a characteristic crop of the Mediterranean area, recognized for its nutritional value and therapeutic properties due to the presence of bioactive components such as polyphenols, inulin, vitamins and minerals. Artichoke is mainly consumed after home and/or industrial processing, and the undersized heads, not suitable for the market, can be used for the recovery of bioactive compounds, such as polyphenols, for cosmetic applications. In this paper, the potential skin anti-age effect of a polyphenolic artichoke extract on endothelial cells was investigated. The methodology used was addressed to evaluate the antioxidant and anti-inflammatory activities and the improvement of gene expression of some youth markers. The results showed that the artichoke extract was constituted by 87% of chlorogenic, 3,5-*O*-dicaffeoylquinic, and 1,5-*O*-dicaffeoylquinic acids. The extract induced important molecular markers responsible for the microcirculation and vasodilatation of endothelial cells, acted as a potential anti-inflammatory agent, protected the lymphatic vessels from oxidative damage by ROS formation, and enhanced the cellular cohesion by reinforcing the tight junction complex. In addition, the artichoke extract, through the modulation of molecular pathways, improved the expression of genes involved in anti-ageing mechanisms. Finally, clinical testing on human subjects highlighted the enhancement by 19.74% of roughness and 11.45% of elasticity from using an artichoke extract cosmetic formulation compared to placebo cream.

## 1. Introduction

*Cynara cardunculus* var. *scolymus* L. Hayek, commonly known as artichoke, is a plant characteristic of the Mediterranean area belonging to the *Asteraceae* family [1]. Europe, and in particular Italy, is the main producer of artichokes, with annual production of 737,591 and 451,461 tons, respectively [2]. The edible portion of artichoke is represented by an immature inflorescence (head) composed of tender bracts, and a tasty receptacle. Artichoke is well known for its nutritional values and therapeutic properties [3] due to the presence of bioactive components such as polyphenols, inulin, vitamins and minerals [1]. Artichoke can also be considered a rich source of vitamin C (10 mg 100 g^−1^ FW), K and Ca (360 and 50 mg 100 g^−1^ FW, respectively), Fe and Zn (47 and 26.2 mg kg^−1^ DW) [4,5,6]. Regarding polyphenols, their concentrations in artichoke can be influenced by several factors, such as genotypes, physiological stages, agro-technical processes, and the different parts considered (receptacle, inner and outer bracts, and leaves) [7]. Instead, the qualitative composition of artichoke polyphenols can be mainly attributed to two phenolic classes: hydroxycinnamic acids and flavonoids. Among the hydroxycinnamic acids, 5-*O*-caffeoylquinic acid (chlorogenic acid), 1,5-*O*- and 3,5-*O*-dicaffeoylquinic acids are the most abundant. Furthermore, apigenin and luteolin (both present as glucosides and rutinosides) are the main flavonoids present in the artichoke [8]. Finally, in some cultivars with violet inner bracts, cyanidin caffeoylglucoside derivatives have also been identified [9]. All these components confer to the artichoke some beneficial and therapeutic characteristics. Several scientific reports have demonstrated the health-promoting properties of the artichoke, such as lipid-lowering, diuretic, anticarcinogenic, anti-HIV, antioxidative, antifungal and antibacterial properties [1,10,11,12]. Positive in vitro and in vivo effects of the artichoke polyphenols on Low-Density Lipoprotein (LDL) oxidation inhibition were reported by D’Antuono et al. [8] and Coinu et al. [13]. Other interesting results on the hypoglycemic effect of the artichoke were reported by Nazni et al. [14] on type 2 diabetic individuals. The authors have recorded a significant reduction of postprandial blood glucose using biscuits enriched with artichoke. Finally, Saénz Rodriguez et al. [15] evaluated the in vivo choleretic activity of an artichoke leaf extract, highlighting the significant increase in bile flow and choleretic effect similar to that produced by standard choleretic drugs.

Artichoke is mainly consumed after home and/or industrial processing. The latter generates large amounts of disposable material (about 80–85% of the total plant biomass), mainly constituted by bracts and stems. This waste is not appropriate for human consumption, and it is used to recover high-molecular-weight inulin [16], for the extraction and recovery of enzymes such as peroxidase [17], and for biofuel production [18]. Artichoke waste is still rich in polyphenols [19,20]; recently, some authors have proposed an eco-friendly procedure to recover polyphenols that employs aqueous ethanol as a solvent and produces a renewable bioenergy [21]. Besides the discussed biomass, there are other artichoke by-products: undersized heads, which are not suitable for the market, can be used for the recovery of bioactive compounds, such as natural antioxidants for food, pharmaceutical, and cosmetic applications [22].

Vascular aging is characterized by modifications of the functionality and the structure of the vessel walls, constituted by endothelial and smooth muscle cells. The circulatory system includes two distinct systems that work in tandem: the cardiovascular circulatory system and the lymphatic circulatory system. While the cardiovascular system is formed of blood vessels, whose inner wall is constituted of endothelial cells (EC), the lymphatic one is a network of lymphatic vessels and lymph capillaries, supported by lymphatic endothelial cells (LEC). Several studies have shown that the ageing process can seriously affect the functionality of both circulatory systems, through the generation of reactive oxygen species (ROS), including nitrate ion reactive derivatives, such as peroxynitrite [23]. Skin functionality is significantly affected by vascular aging, as damages or alterations to the blood vessel wall architecture limit the correct delivery of nutrients to the skin cells, including fibroblasts, which are the most important cellular component of the dermis [24]. In addition, the aged lymphatic vessels fail to remove the excess of fluid, waste products and toxins from the intracellular spaces, compromising the overall appearance of the skin and accelerating the physiological aging process [25]. Indeed, with the advancement of age, the lymphatic endothelial barrier integrity and function tend to decline because the cell membranes lose their retention capacity, causing excessive leakage of fluid and proteins from the vessels to the interstitial space [26].

A good dual strategy to fight the skin degenerative process occurring during senescence is represented by both the enhancement of the membrane permeability in blood endothelial cells, thus nutrient delivery to skin cells, and the reduction of the membrane leakage in the lymphatic endothelial cells.

The well-documented beneficial properties of artichoke polyphenols [1] are mainly related to chlorogenic acid as a free radical scavenger and UV protector, and to dicaffeoylquinic acids as anti-inflammatory agents [27,28,29,30]. This starting knowledge prompted us to investigate the potential role of an artichoke ethanolic extract, recovered from undersized heads, in promoting the endothelial cell functions and stimulating the gene expression of some youth associated markers, with the main goal of characterizing it as cosmetic anti-age ingredient.

## 2. Results and Discussion

### 2.1. Chemical Characterization of Artichoke Ethanolic Extract

In Table 1, the amount of the main polyphenols present in the water/ethanolic extract of undersized artichoke heads, identified by HPLC-DAD analysis is reported. In particular, the results highlight the presence of chlorogenic, 3,5-*O*-dicaffeoylquinic, and 1,5-*O*-dicaffeoylquinic acids, as the main constituents in artichoke heads. Their relative abundance is 87%, as reported by other authors [1,8,31]. Moreover, other mono and di-caffeoylquinic acids, and in particular 1-*O*-caffeoylquinic acid, 3-*O*- caffeoylquinic acid, 1,4-*O*-dicaffeoylquinic acid, 4,5-*O*-dicaffeoylquinic acid, and 3,4-*O*-dicaffeoylquinic acid, were also identified in the extract. Finally, the flavonoid apigenin-7-*O*-glucoside was also identified, according to reports by other authors [31]. To obtain a powder suitable for the cellular bioassays, the artichoke ethanolic extract (AEE) was lyophilized, and the polyphenol composition was verified, confirming the presence of all the identified compounds.

### 2.2. Artichoke Extract Activity in Endothelial Cells

On the basis of the chemical analysis and literature data, we investigated the potential activity of the AEE to promote endothelial cell functions. As explained in the introduction, vascular ageing is one of the primary causes of skin structure alterations, as the endothelial cells forming the capillaries are responsible for the correct exchange of nutrients and oxygen between the skin cells and the blood. To test the ability of the AEE to affect microcirculation and vasodilation in endothelial cells, gene expression analyses of the Vascular Endothelial Growth Factor (VEGF), the Endothelin 1 (ET-1), and the endothelial Nitric Oxide Synthase (eNOS) were conducted. Although VEGF is well known for its role in angiogenesis and wound repair [32], it has also been associated with an increase of capillary permeability in the skin [33]. Analogously, ET-1, a signal peptide produced by the cells, enhances trans-endothelial permeability when binding to the specific transmembrane receptors [34], and is able to activate eNOS expression, which in turn modulates the vascular tone by Nitric Oxide (NO) synthesis and release [35]. As shown in Table 2, analogously to the positive control TGF-β, the AEE increased the expression of all three analyzed genes in human endothelial cells in a dose-dependent manner with respect to the untreated control, suggesting a potential effect of vasodilation and capillary permeation, and thus an enhancement of blood microcirculation, in the skin. Similar results were obtained with other classes of polyphenols, like resveratrol and quercetin at physiological concentrations (0.1 μM), on the same cellular lines. The authors concluded that, although the antioxidant properties of these compounds are well known, it is not clear if their capacity to influence the gene expression in endothelial cells can be directly correlated with their chemical structures or antioxidant capacity [36].

### 2.3. Artichoke Extract on Nitric Oxide Production in Macrophages

**Figure 1:**
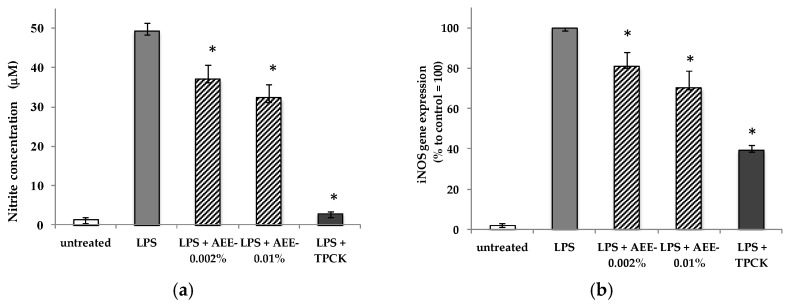
Protection against inflammation. The levels of NO production (**a**) and iNOS gene expression (**b**) were measured in RAW 264.7 macrophages treated with AEE before addition of bacterial liposaccharide LPS. TPCK was used as positive control. All determinations were conducted in triplicate and results are expressed as mean ± SD. * *p* < 0.05.

To exclude the triggering of potential inflammatory reactions in the skin due to an excess of NO production, we treated macrophages, the main cells responsible for the initiation of the inflammatory cascade in the skin, with the AEE and measured the amount NO released by the cells as pro-inflammatory mediator [37]. As shown in Figure 1a, under stress conditions caused by the lipopolysaccharide (LPS), the production of NO was attenuated by 24.5% and 34.7% by the AEE, at concentrations of 0.002% and 0.01%, respectively. As expected, the NO synthesis was strongly inhibited by the *N*-p-tosyl-l-phenylalanine chloromethyl ketone (TPCK) (10 µM), a commercially available anti-inflammatory drug used as a positive control in the assay [38]. These results agree with previous studies [39] performed on activated macrophages using different classes of polyphenols, in particular flavonoids. The authors indicated that the anti-inflammatory effects recorded were dose-dependent, although the flavonoid concentrations used were higher than those reported in this study. The inhibition of NO production caused by the AEE was confirmed by the gene expression analysis of the inducible Nitric Oxide Synthase (iNOS) (Figure 1b), enzyme involved in the synthesis of NO after an inflammatory stimulus, suggesting that the extract produced a dual effect on NO production in the endothelial cells and macrophages, and may also work as a potential anti-inflammatory ingredient for the skin.

### 2.4. Artichoke Extract Activity in Lymphatic Endothelial Cells

**Figure 2:**
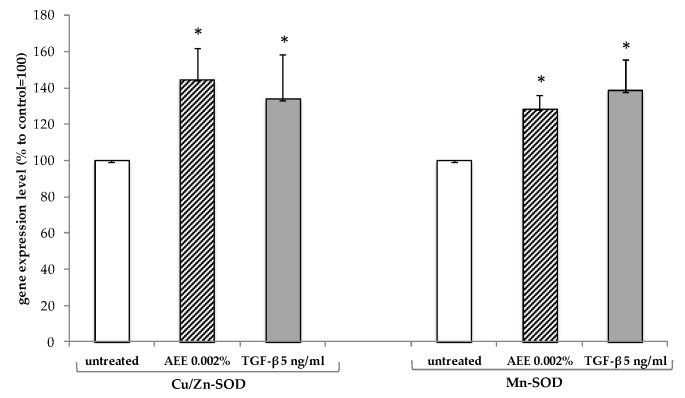
Effect of AEE on Superoxide Dismutases (SOD) in lymphatic endothelial cells. The gene expression level of the Cu/Zn-SOD and Mn-SOD was measured in HDLEC incubated with AEE or TGF-β, used as positive control. All determinations were conducted in triplicate and the results are expressed as mean ± SD. * *p* < 0.05.

**Figure 3:**
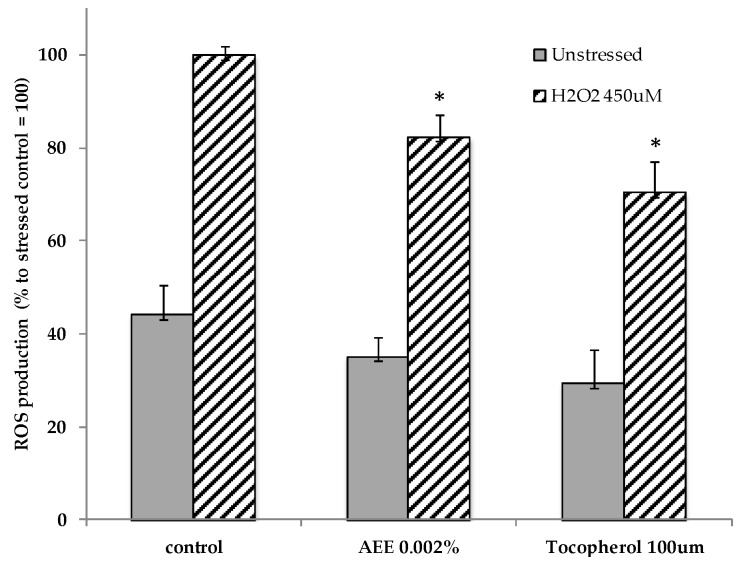
Protection against oxidative stress. The amount of ROS production was measured in HDLEC treated with 0.002% of AEE (% of Artichoke Ethanolic Extract powder used in the assay, *w*/*v*), before and after oxidative stress induced by 450 μM H_2_O_2_. α-Tocopherol was used as positive control in the assay. All determinations were conducted in triplicate and the results are expressed as mean ± SD. * *p* < 0.05 compared to stressed control, set as 100%.

The other important components of the circulatory system are the vessels formed by the lymphatic endothelial cells, which, differently from the blood endothelial cells, are responsible for the lymph flow within the vessels and for the removal of waste products from the cell interstitial spaces [40]. To investigate on the capacity of the AEE to affect lymphatic endothelial cell functionality, we measured the induction of the genes involved in the oxidative stress protection and those involved in the formation of the tight junctions (TJs) in HDLEC cellular line. TJs are protein complexes with key roles in the maintenance of a tight cohesion among the cells that form the lymphatic vessels [41]. Preliminary tests related to toxicity and biological activity indicated that the optimal concentration of the AEE to use on the HDLEC, to detect the highest response in terms of gene expression after 4 h, was 0.002%. The treatment with 0.002% AEE stimulated the gene expression of both the cytosolic and the mitochondrial Superoxide Dismutase (Cu/Zn-SOD; Mn-SOD) by about 45% and 29%, respectively, suggesting anti-oxidant protection activity against ROS formation (Figure 2). The protective effect was also determined by measuring the lipid peroxide production in the HDLEC after oxidative stress in absence and in the presence of 0.002% AEE. Lipid peroxides are unstable compounds that are formed at the level of the cell membranes in response to strong oxidative stress and give rise to soluble oxidant species that rapidly diffuse throughout the cytoplasm. The fluorescent signal, given by the interaction of membrane ROS with the probe, was significantly reduced by the AEE treatment, similarly to the positive control tocopherol, indicating a protective effect of the extract towards oxidative stress induced by H_2_O_2_ in lymphatic cells (Figure 3). Moreover, AEE induced the gene expression of two of the most relevant cell adhesion factors constituting the TJ complex, Claudin-5 (Cla-5) and Zona Occludens 1 (ZO-1) [42]. Compared to the untreated control, the cells treated with the AEE had a higher level of mRNA corresponding to the two analyzed genes, suggesting a reinforcement of the tight junction complex, and thus higher cohesion among the lymphatic cells (Table 3). The effect of the AEE was also significant on the gene expression of another gene involved in the lymphatic cell membrane trafficking, the receptor for hyaluronan lymphatic vessel endothelial hyaluronan receptor 1 (LYVE-1), extensively studied for its involvement in the intracellular signaling pathways for endothelial junctional retraction and lymphatic endothelial proliferation [43] (Table 3).

**Figure 4:**
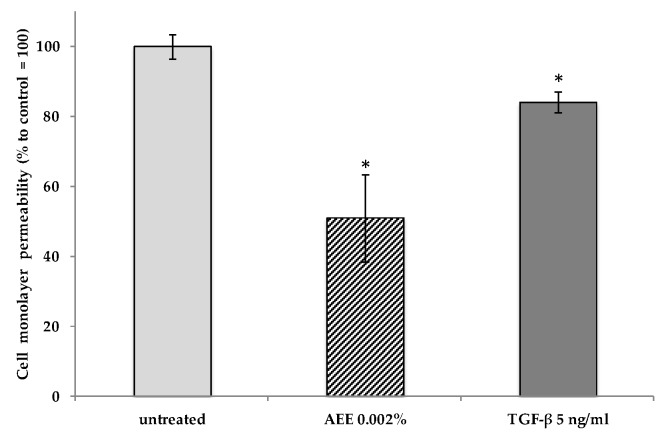
Intercellular permeability assay. HDLEC were previously treated with the AEE and TGF-β (positive control) and then exposed to the fluorescent compound fluorescein. The amount of fluorescein passed through the cell monolayer was measured by a spectrophotometer. All determinations were conducted in triplicate and the results are expressed as mean ± SD. * *p* < 0.05 compared to the untreated control.

The increase of cell cohesion was validated by a cell permeability assay, where the amount of a fluorescent probe passing through a cell monolayer comprising tightly adherent lymphatic cells was evaluated by using trans-well plates. As shown in Figure 4, treatment with AEE produced a significant decrease in the amount of fluorescein passed from the top to the bottom of the well through the cell monolayer, indicating an increase in tight junction formation and cell cohesion. An analogous effect was also evident in the sample treated with TGF-β, used as a positive control in the assay for its role in enhancing TJ formation in different cell types [44]. All these results indicated that the compounds contained in the artichoke extract induced a reinforcement of the lymphatic cell physical barrier by TJs, thus preventing solutes and water from passing freely through the paracellular spaces among the cells.

### 2.5. Artichoke Extract Activity in Clinical Tests

**Figure 5:**
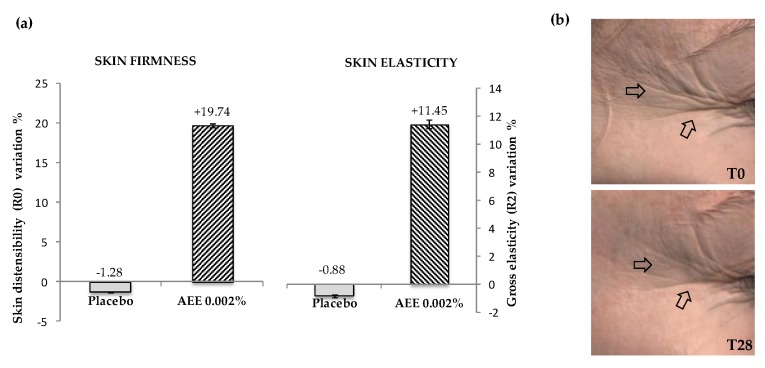
Clinical tests on human skin. 20 female volunteers were treated for 28 days either with a cream containing 0.002% of AEE or with a placebo cream without the extract. The effects on skin firmness and elasticity were measured by a Cutometer and expressed in the graph as R0 and R2 parameters (**a**). The effects on wrinkle depth were visualized by a 3D camera and photographed (**b**). The error bars indicate standard deviations, * *p* < 0.05 compared to placebo control.

On the basis of the in vitro data on cell cultures, AEE was tested for its capacity to ameliorate skin firmness and elasticity. Indeed, an improvement of the vascular functions in the skin, in terms both of blood microcirculation and lymph drainage, may significantly affect skin quality, slowing down the degenerative process leading to wrinkle formation and loss of elastic fiber functionality [25,45]. For this purpose, 20 female volunteers between 35 and 55 years of age presenting sagging face applied a cream containing 0.002% of AEE for 28 days on half of their face, while on the other half, they applied a placebo cream without the extract. The effects on wrinkle depth and elastic properties were measured by an Antera 3D camera, which made it possible to capture high-resolution images to document the changes over time and treatments, providing a detailed view of the skin in 2 and 3 dimensions in order to evaluate the texture and roughness. The results, reported in Figure 5a, indicated that the artichoke extract improved skin roughness and elasticity by 19.74% (*p* < 0.05) and 11.45% (*p* < 0.05), respectively, in treated individuals, compared to those treated with a placebo cream only.

## 3. Materials and Methods 

### 3.1. Reagents 

Extraction and chromatography solvents, ethanol (EtOH), methanol (MeOH), and acetic acid (AcOH) were HPLC certified. The polyphenol standards used in this study, such as chlorogenic acid, 3-*O*-caffeoylquinic acid, 1,5-*O*-dicaffeoylquinic acid, 3,4-*O*-dicaffeoylquinic acid, 3,5-*O*-dicaffeoylquinic acid and 4,5-*O*-dicaffeoylquinic acid, were purchased from PhytoLab GmbH & Co. KG (Vestenbergsgreuth, Germany). *N*-p-tosyl-l-phenylalanine chloromethyl ketone (TPCK), lipopolysaccharide (LPS), α-tochopherol and hydrogen peroxide solution (H_2_O_2_) were purchased from Sigma Aldrich Co. (Milano, Italy). Human TGF-β recombinant protein, 4,4-difluoro-3a,4a-diaza-s-indacene (BODIPY), dextran fluorescein 3000 MW and the Griess reagent [*N*-(1-naphthyl)ethylenediamine and sulfanilic acid] were purchased from Invitrogen-Life Technologies (Carlsbad, CA, USA).

### 3.2. Raw Materials

The samples used in this study were extracts of by-products of artichoke heads (*Cynara cardunculus* (L.) subsp. *scolymus* Hayek cv. Violetto di Provenza) that were not suitable for the market due to non-compliant features, like being undersized, picked, etc. They were supplied by the local producer “Società Agricola Protopapa a r.l.”, Brindisi, Italy, and stored at 4 °C until analysis.

### 3.3. Preparation of Artichoke Polyphenol Extract

Before processing, the raw material was cleaned, and all the damaged external bracts were manually removed. Furthermore, artichoke heads were homogenized (knife mill Grindomix GM300—Retsch, Germany) in a mixture of food grade solvents (EtOH:H_2_O; 80:20 *v*/*v*) with a ratio of 1:5 *w*/*v*, and were then extracted by maceration at room temperature for 1 h. The extract was filtered through a Whatman 1 paper, and the organic solvent was evaporated under vacuum (rotary evaporator IKA^®^-Werke GmbH & Co. KG, Germany). The water was additionally removed by lyophilization (VirTis AdVantage Pro–SP Scientific, Gardiner, NY, USA), until a fine powder was obtained. Finally, the powder was dissolved in water at a concentration of 10% *w*/*v* to be used in the chemical analyses or the bioassays, in order to realize two different final concentrations in the reaction solution (0.002 and 0.01%).

### 3.4. HPLC-DAD Analysis

HPLC analysis was carried out using Agilent 1260 Infinity System (Agilent Technologies, Inc., Santa Clara, CA, USA), equipped with a HiP Degasser, binary pump, TCC Thermostat, Diode Array Detector and Agilent Open Lab Chem Station Rev C.01.05 software. The chromatographic separation was performed at a flow rate of 1 mL/min, with Luna C-18 column (5 μm; 4.6 × 250 mm, Phenomenex Torrance, CA, USA), thermostated at 40 °C. The polyphenol detection was monitored using a photo diode array detector at three preferred wavelengths: 280 nm, 325 nm, and 360 nm. Polyphenols were identified using the spectra and retention times of the pure available standards. In addition, the identification of 1-*O*-caffeoylquinic and 1,4-*O*-di caffeoylquinic acids was performed based on the spectrum analysis and classification reported in Lattanzio et al. [1]. Finally, chlorogenic acid and 3,5-*O*-dicaffeoylquinic acid were used for the quantification of mono-caffeoylquinic acids and dicaffeoylquinic acids, respectively. The results were expressed as mg/100 g of fresh weight for the artichoke ethanolic extract, and as mg/g powder for the lyophilized sample.

### 3.5. Cell Cultures

Human Umbilical Vein Endothelial Cells (HUVEC) (Lonza Walkersville, MD, USA) were grown in Endothelial Basal Medium (EBM), containing 2% Fetal Bovine Serum (FBS), Bovine Brain Extract (BBE), hydrocortisone, human Epidermal Growth Factor (hEGF), Gentamicin and Ascorbic acid (EGM Lonza). Human Dermal Lymphatic Endothelial Cells (HDLEC) (PromoCell, Heidelberg, Germany) were grown in EBM, containing 5 ng/mL hEGF, 10 ng/mL basic Fibroblast Growth Factor, 20 ng/mL Insulin-like Growth Factor, 0.5 ng/mL vascular endothelial growth factor, 1 μg/mL ascorbic acid and 0.2 μg/mL hydrocortisone (MV2 PromoCell). Murine RAW 264.7 macrophages were purchased from the European Collection of Cell Cultures (ECACC; Health Protection Agency Culture Collection, Porton Down, UK) and were grown in Dulbecco’s Modified Eagle Medium (DMEM Gibco), containing 2 mM L-glutamine, 4.5 g/L glucose, 1 mM sodium pyruvate, and 1.5 g/L sodium bicarbonate, supplemented with 10% FBS, under 5% CO_2_ humidified atmosphere at 37 °C.

### 3.6. Reactive Oxygen Species Determination 

To measure membrane ROS production, 1.3 × 10^4^ HDLEC were seeded in 96-well plates for 20 h and then incubated for 2 h with the AEE extracts, or with 100 μM tocopherol used as positive control. Subsequently, they were washed in PBS and incubated with the dye C11-BODIPY at 37 °C for 30 min. After an additional wash in PBS, the cells were stressed with 450 μM H_2_O_2_ and incubated for 1 h. The fluorescence of the samples was thus measured at 535 nm (excitation 490 nm), using the instrument EnVision (PerkinElmer).

### 3.7. Gene Expression Analysis in HDLEC and HUVEC

HUVEC and HDLEC were grown in 6-well plates in EBM up to a density of 1.5 × 10^4^ per well for 24 h. The cells were then incubated for 4 or 16 h at 37 °C, 5% CO_2_, with the artichoke extract or 2.5 ng/mL TGF-β, used as positive control. Total RNA was extracted with the GenElute Mammalian Total RNA Purification Kit (Sigma-Aldrich Co., Milano, Italy) and treated with DNAse I at 37 °C for 30 min, according to the manufacturer’s instructions. cDNA was synthesized from 1 μg of RNA using the RevertAidTM First Strand cDNA Synthesis (ThermoFisher Scientific, Waltham, MA, USA) and RT-PCR was conducted by using gene specific primers and the Quantum RNATM 18S internal standard (AmbionTM). The amplification reactions followed this general protocol: 2 min at 94 °C; 35 cycles at 94 °C for 30 s, at the annealing temperature (specific for each gene) for 30 s, and at 72 °C for 30–60 s, with a 10 min final extension at 72 °C. The obtained amplification products were run on a 1.5% agarose gel, and the bands were quantified by the Geliance 200 Imaging system (Perkin Elmer, Waltham, MA, USA). The amplification band corresponding to each amplified gene was normalized to the amplification band corresponding to the 18S. The obtained measure was converted into a percentage value by setting the untreated control as 100%. All the semi-quantitative RT-PCRs were repeated three times to ensure quality of reproduction. The following primers were used in the reactions:

hVEGF fw: TGCATTGGAGCCTTGCCTTG

hVEGF rv: GAAGATGTCCACCAGGGTCT

hEt-1 fw: TGTCTACTTCTGCCACCT

hEt-1 rv: TCACCAATGTGCTCGGTTGT

heNOS fw2: GAAGATCTCCGCCTCGCTC

heNOS rv2: GGACACCACGTCATACTCAT

Sirt1-F: ATACCCCATGAAGTGCCTC

Sirt1-R: CGTCATCTTCAGAGTCGTA

Sirt3Fw: TTGGCCAAGGAGCTGTAC

Sirt3Rv: TGGCAAAAGGCTCCACCT

Sirt6-F: ATCACGCTGGGTACATCG

Sirt6-R: ACCTCGTCAACGTAGCCAT

GDF11 Fw CAGTCTACCTGCAGATCT

GDF11 Rv CCAAATGGGTATGCGGATA

### 3.8. Nitric Oxide Assay and iNOS Expression in Macrophages

For gene expression, RAW 264.7 murine macrophages (1.5 × 10^5^ cells/well) were grown at 37 °C in DMEM medium containing 10% FBS for 24 h in 6-well plates. Cells were treated for 2 h with the extracts or 10 µM TPCK, used as positive control, and then stressed by the inflammatory agent LPS (1 µg/mL) for 4 h. The RNA was extracted and processed as previously described. The primers used in the PCR reactions were: iNOS-F (5′acaacatcctggaggaagtg3′) and iNOS-R (5′tccatgcagacaaccttgg3′). The NO concentration was determined in macrophages, seeded at a concentration of 1.5 × 10^5^ cells/well in 96-well plates for 24 h, and pre-treated with the extracts or with 10 µM TPCK for 2 h, before the treatment with 1 µg/mL LPS for 18 h. The amount of NO, converted into nitrite, was calculated by adding Griess reagent [solution of *N*-(1-naphthyl)ethylenediamine and sulfanilic acid, Invitrogen-Life Technologies], and after 30 min measuring the absorbance at 540 nm by the multiwell-plate reader, Victor3 (Perkin Elmer).

### 3.9. Permeability Assay

HDLEC were seeded in a trans-well plate with 3 μM pores at a density of 1 × 10^6^ per well. 48 h later, the cells were incubated for 6 h at 37 °C, 5% CO_2_, with the extracts or with 0.4 μg/mL of TGF-β, used as positive control. After 24 h, FITC-Dextran 10 μg/mL (3kDa) was added to the cells in the upper compartment, and the amount of dextran passed into the lower compartment was monitored every 10 minutes, by measuring the fluorescence at 535 nm of an aliquot of the solution each time.

### 3.10. Clinical Tests

To analyze the AEE efficacy on the skin complexion, a clinical test was performed. Parameters analyzed were detected through the use of a Cutometer MPA 580 instrument (Courage + Khazaka electronic GmbH, Cologne, Germany) and a 3D Antera camera (Miravex, Dublin, Ireland). The Cutometer allows the quantification of parameters such as skin distensibility (R0) and skin overall elasticity (R2), while the Antera camera captures images in 3D based on optical advanced technology, thus allowing the evaluation of overall skin texture and wrinkle depth (mm). Twenty female volunteers were recruited, ranging from 35 to 55 in age and presenting sagging face, who applied a cosmetic formulation containing 0.002% AEE for 28 days on half of their face, while on the other half they applied the same formula not containing AEE (placebo). The study was conducted in wintertime, and the patients were asked not to expose their face to direct sunlight. Informed consent was obtained from the subjects before entering into the study, which was performed by Eurofins Biolab (Rome, Italy) according to the principles of medical ethics in clinical research in the Declaration of Helsinki (June 1964) and its successive amendments, and approved by the Directive of the European Parliament and Council 2001/20/EC. All the obtained data after 28 days of bi-daily application were subjected to a statistical analysis using Student’s *t*-test for repeated measures.

### 3.11. Statistical Analysis

Each value in the graphs or tables is expressed as mean ± Standard Deviation (SD) of three independent experiments, conducted in triplicate. One-way ANOVA was used for multiple comparisons using the Microsoft Excel program. A value of *p* < 0.05 was considered significant.

## 5. Conclusions

In this work, the effect of a polyphenolic artichoke extract in promoting endothelial cell functions was investigated through different mechanisms. In particular, AEE improved the microcirculation and vasodilatation of endothelial cells, worked as potential anti-inflammatory agent by the inhibition of NO production in both endothelial cells and macrophages, protected the lymphatic vessels from oxidative damage by ROS formation and enhanced the cellular cohesion by reinforcement of the TJ complex, also reducing the cellular monolayer permeability. Finally, in vivo studies on human subjects with sagging face reported an improvement in roughness and elasticity with application of the artichoke extract cosmetic formulation compared to the placebo cream. The results reported in this study can provide useful information on how the polyphenolic components of AEE can improve skin roughness and elasticity by inhibiting the vascular aging process, thus acting as a protective ingredient for both endothelial and lymphatic cells. This effect could be performed directly by their antioxidant or anti-inflammatory properties, and indirectly, via the modulation of molecular pathways that improve the expression of genes involved in anti-ageing mechanisms. Moreover, the here-presented study gives good suggestions regarding the possibility of reusing vegetable by-products, such as undersized artichoke heads, for cosmetic and nutraceutical applications.

## Figures and Tables

**Table 1:** Polyphenol composition of the artichoke head extract before and after lyophilization, performed by HPLC-DAD analysis.

Phenolic Compounds	Artichoke Extract	
	Fresh (mg/100 g FW)	Lyophilized (mg/100 g powder)
1-*O*-caffeoylquinic acid	3.4 ± 0.3	0.8 ± 0.1
3-*O*-caffeoylquinic acid	2.1 ± 0.2	0.5 ± 0.1
Chlorogenic acid	236.2± 18.2	57.3 ± 1.4
1,4-*O*-dicaffeoylquinic acid	5.0 ±0.4	1.3 ± 0.1
4,5-*O*-dicaffeoylquinic acid	8.2 ± 0.7	2.0 ± 0.1
3,5-*O*-dicaffeoylquinic acid	160.1 ± 13.1	39.8 ± 1.0
1,5-*O*-dicaffeoylquinic acid	201.3 ± 17.9	50.1 ± 1.2
3,4-*O*-dicaffeoylquinic acid	21.7 ± 1.9	5.4 ± 0.1
Apigenin-7-*O*-glucoside	19.9 ± 1.8	4.8 ± 0.4
Total phenolics	657.8 ± 56.3	162.0 ± 3.9

Data are expressed as mean ± SD of three independent experiments; FW = fresh weight.

**Table 2:** Effects of AEE on microcirculation and vasodilation in endothelial cells. The gene expression level of VEGF, ET-1 and eNOS was measured in HUVEC incubated with two different concentrations of AEE (% of extract powder used in the assay, weight/volume) or TGF-β (2.5 ng/mL), used as positive control. The measures reported indicate the increases of gene expression level (expressed as %) of the treated samples compared to the untreated controls.

Analyzed Gene	AEE 0.002%	AEE 0.01%	TGF-β 2.5 ng/mL
**VEGF**	11.1 ± 6.1 *	31.4 ± 7.4 *	47.1 ± 11.2 *
**ET-1**	33.5 ± 14.3 *	43.9 ± 9.4 *	26.1 ± 7.4 *
**eNOS**	22.2 ± 4.7 *	59.4 ± 2.3 *	10.6 ± 2.1 *

Notes: AEE = Artichoke Ethanolic Extract; VEGF = Vascular Endothelial Growth Factor; ET-1 = Endothelin 1; eNOS = endothelial Nitric Oxide Synthase, HUVEC = Human Umbilical Vein Endothelial Cells; TGF-β = transforming growth factor-beta. All determinations were conducted in triplicate and the results were expressed as mean ± SD. * *p* < 0.05, compared to the untreated control.

**Table 3:** Effects of AEE on tight junction proteins in lymphatic endothelial cells. The gene expression levels of Cla-5, ZO-1 and LYVE were measured in HDLEC incubated with two different concentrations of AEE (% of extract powder used in the assay, weight/volume) or TGF-β (5 ng/mL), used as positive control. The measures reported indicate the increases of gene expression level (expressed as %) of the treated samples compared to the untreated controls.

Analyzed Gene	AEE 0.002%	TGF-β 5 ng/mL
**Cla-5**	39.7 ± 6.8 *	42.7 ± 6.5 *
**ZO-1**	46.9 ± 12.1 *	42.5 ± 7.4 *
**LYVE**	44.7 ± 7.7 *	28.1 ± 2.3 *

Notes: AEE = Artichoke Ethanolic Extract; Cla-5 = Claudin-5; ZO-1 = Zona Occludens 1; LYVE = Lymphatic vessel endothelial hyaluronan receptor 1; HDLEC = Human Dermal Lymphatic Endothelial Cells. All determinations were conducted in triplicate and the results are expressed as mean ± SD.* p < 0.05 compared to the untreated control.
